# Investigation of Surface Roughness and Predictive Modelling of Machining Stellite 6

**DOI:** 10.3390/ma12162551

**Published:** 2019-08-10

**Authors:** Jan Valíček, Jan Řehoř, Marta Harničárová, Miroslav Gombár, Milena Kušnerová, Jaroslava Fulemová, Alena Vagaská

**Affiliations:** Faculty of Mechanical Engineering, Regional Technology Institute, University of West Bohemia, Univerzitni 8, 306 14 Pilsen, Czech Republic

**Keywords:** Stellite 6, longitudinal turning, prediction of topographic parameters, surface roughness

## Abstract

The aim of the paper was to examine the influence of cutting conditions on the roughness of surfaces machined by longitudinal turning, namely of surfaces coated with Stellite 6 prepared by high-velocity oxygen fuel (HVOF) technology and applied onto a standard structural steel substrate. From the results of measurements of the cutting parameters, a prediction model of the roughness parameters was created using mathematical and statistical methods. Based on a more detailed analysis and data comparison, a new method for prediction of parameters of longitudinal turning technology was obtained. The main aim of the paper was to identify the mutual discrete relationships between the substrate roughness and the machining parameters. These were the feed rate *v_c_* (m·min^−1^), in the case of turning and milling, and the feed rate *f* (mm·rev^−1^) and the depth of cut *a_p_* (mm). The paper compared and verified two approaches of this method, namely the mathematical statistical approach, the analytical approach and measured dates. From the evaluated and interpreted results, new equations were formulated, enabling prediction of the material parameters of the workpiece, the technological parameters and the parameters of surface quality.

## 1. Introduction

Advanced machining is a highly sophisticated technological process based on stock removal from a workpiece. The physical nature of the stock material is that the action of external energy, especially mechanical, disrupts the interconnections between the elementary particles of the material from which the chip is gradually separated, that is, the material in the form of smaller particles. The result of machining is a component of the required geometric dimensions with the required dimensional accuracy and surface quality, functional and with a sufficient service life in the given machinery equipment. Todd et al. [1] have classified different manufacturing processes as property modification processes and geometry modification processes. 

The technologically more demanding type of material machining, from the viewpoint of achieving the desired surface properties, is machining of coatings. The filler material for welding is chosen in connection with the welding technology. Durable materials are usually divided by the type of matrix, i.e., materials with iron, cobalt and nickel matrices. From the viewpoint of the physical properties of the material, i.e., in particular, the thermal expansion of the basic and filler material, the values of the coefficients of thermal expansion of both materials must be approximately similar in order to prevent the creation of cracks during solidification. A wide range of iron-based materials exists at present on the market. Materials with high contents of carbide have lower impact strength, but excellent abrasion resistance. Martensitic and austenitic alloys have better impact resistance and sufficient abrasion resistance; therefore, they are used to harden surfaces. Kracke and Allvac [2] described the history of the superalloy industry, and their work addressed the present and future uses of superalloys that are possible thanks to their properties. Nickel and cobalt alloys are used for high-temperature applications. Significant progress has been achieved in developing new materials and in machine tool design. Rivin [3] provided a survey on six essential subjects related to tooling structures, namely, the influence of machining system parameters on tool life and process stability, stiffness and damping of tools, tool–tool holder interfaces (tool clamping devices), modular tooling, tool–machine interfaces and tool balancing for high-speed machine tools. During the cutting process, the division of material is realised by a cutting wedge. Cutting is, in principle, the stock removal of the cut-off layer by a cutting edge at a certain cutting speed, caused by the corresponding cutting force and cutting resistance, which are particularly significant in the case of materials difficult to machine. Due to the considerable cutting resistance and the corresponding strong friction, considerable heat is also generated, and the mechanisms of wear of the tool cutting edge are activated. The associated result of machining is then a surface of the workpiece that can be characterised by the topography and integrity of the surface layer explicitly required by the use. The term machinability is often used for determining how easily a material can be machined. Davim [4] described in his book the level of difficulty involved in machining a material, or multiple materials, with the appropriate tooling and cutting parameters. A combination of specific operating conditions with the condition and physical properties of the machined workpiece influence and determine machinability. The surface finish left on the workpiece during a machining operation is used to determine the rate of machinability of a metal. The surface roughness is one of the first visible factors of the quality of the product. 

### 1.1. Investigation of Surface Roughness

Valíček et al. [5] dealt with the surface roughness and machinability of different materials through the identification of surfaces after machining in a non-contact manner. They presented a new, modified measurement method, where the scanned intensity distribution at the defocusing plane gives the information necessary to assess the second derivatives, and thus, surface functions, which can be used to determine the groove curvatures of the real surface morphology. 

Hloch and Valíček [6] proposed a new way of classifying morphologically different zones, based on the quantitative parameters *Ra* and *Rz* as a base for optimisation and control of different technologies, such as abrasive water-jet cutting. They found a close connection between the geometric condition of the surface and the physical–mechanical parameters of materials machined by abrasive water-jet, including tensometric changes due to external stress in the elastic and plastic regions that can be easy to use for control of the process. Valíček et al. [7,8] have been awarded American and Czech patents covering the design of technology for the cutting of materials by abrasive water-jet using a constant of cuttability, which was derived from the surface deformation parameters. Harničárová et al. [9] presented an approach based on the integrity of the system (technology parameters–tool–material–surface topography) and parameters and interrelations of selected geometric elements that are measurable on the surface of the final cut wall. Their solution enables the prediction of process functions of the technological process for any material, including technological parameters, and an interactive modelling of the technological process of laser cutting.

Results of recent investigations into the surface roughness generated during turning have shown that many machining parameters which control the quality of the surface must be taken into account. The most important are: depth of cut, speed of cut, feed rate, vibration and tool geometry. Machined surfaces have then been analysed in order to find out the most suitable model for the prediction and optimisation of the turning process [10,11,12,13,14,15]. Benardos and Vosniakos performed a comprehensive review of the prediction of surface roughness, considering the various methodologies and approaches [16]. They described many aspects and problems that researchers face in connection with surface roughness modelling. 

Machining is the most extensive area for the application of scientific achievements and for the research of methods that can be used for the measurement and control of surface quality. Knowledge of the topography of surfaces and their classification is essential for different machining processes. 

Current materials research is focused on the applications of available materials and the creation of new materials with desired properties. Materials are generally represented by ceramics, metals and polymers. Each class of materials is the subject of intense interest from many researchers, who are conducting systematic investigations regarding the structure, properties and performance of the material. Generally, they are trying to find answers to the following three essential criteria that are used for the selection of machining parameters: the minimum production cost criterion, the minimum production time and the maximum production rate criterion. One material with specific properties used in industry is Stellite. Stellite is the most widely used of the wear-resistant cobalt-based alloys. 

### 1.2. Stellite Alloy and High-Velocity Oxygen Fuel (HVOF) Technology

The Stellite alloy was invented in 1900 by American metallurgist Haymer E. Haynes as a substitute for silver. In 1907, the company Deloro Smelting and Refining was founded by M. J. O’Brien and E. Haynes in the Canadian village of Deloro. When Haynes began in 1912 to focus on nickel-based components, O’Brien founded the company Deloro Stellite for the production of cobalt-based alloys. Nowadays, this company is recognised worldwide as a renowned producer of approximately 60 types of alloys that contain 4 to 6 of the most significant elements: cobalt, molybdenum, chromium, nickel, tungsten, iron, boron, aluminium, manganese, carbon, phosphorus, silicon, sulphur and titanium in such ratios that the alloys are always characterised by non-magnetism, hardness, toughness and high corrosion resistance.

Stellite 6 is a material based on a cobalt–chromium matrix; it is characterised by high wear resistance and excellent chemical and corrosion resistance over a wide range of temperatures. Chromium, tungsten and molybdenum provide to this alloy resistance to cavitation, abrasion and erosion. The combination of cobalt and chromium contributes to a high melting point that allows the use of this alloy for the production of cutting tools. Due to its high hardness, it is difficult to machine the weld deposits during finishing operations; machining of this material requires the use of specific high-performance machining equipment, which most often grinds material, while cutting is used only in exceptional circumstances. 

The high-velocity oxy-fuel (HVOF) processes are a group of thermal spraying techniques, which are widely used to protect components against corrosion or wear. Sidhu et al. [17] studied the application of Stellite 6 metallic coatings on Ni- and Fe-based superalloys by HVOF process for hot corrosion applications. Coatings had high hardness values in the range of 800–900 Hv, and the porosity of coatings was less than 2%. There have only been a small number of papers published on HVOF-sprayed Stellite 6 coatings. Jegadeeswaran et al. [18] dealt with the high-temperature hot corrosion behaviour of HVOF-sprayed Stellite 6 coatings on gas turbine alloys in a molten salt (Na_2_SO_4_—50% V_2_O_5_) environment at 800 °C, and compared it with uncoated SuperCo-605 and MDN-121. They found a noticeable improvement in corrosion resistance and corrosion behaviour when using sprayed Stellite 6 coatings. 

Nowadays, machinability of cobalt-based alloys is an important research topic for researchers, due to the difficulties of such machining. Several authors have studied the problem of machining of Stellite. Shao et al. [19] made attempts to investigate the machinability of Stellite alloys, and focused on the wear mechanisms and failure modes of the uncoated carbide cutting tools YG610 (K01-K10) and YT726 (K05-K10/M20), and coated tool (SNMG150612-SM1105) when turning. They chose Stellite 12, with a higher hardness than other series of Stellite alloys, and, during turning, an extremely high temperature was reached in the cutting areas in a very short time. It significantly affected the strength of the cutting edge. Zaman et al. [20] presented a general review of the machinability of cobalt-based and cobalt–chromium–molybdenum alloys. They confirmed that studies focused on cutting temperature, chip formation, surface integrity, residual stresses and wear mechanisms were still lacking and provided a great opportunity for future research, especially in the case of high-speed machining. Hasan et al. [21,22] performed experimental research on cutting parameters for turning operations of Stellite 6 using coated carbide inserts. It was revealed that coated carbide inserts with a medium-size nose radius performed better with respect to hardness changes and heat generation, producing minimum phase changes on machined surfaces of Stellite 6. Saidi et al. [23] also dealt with the problem of machinability of Stellite 6. The effects of the turning conditions of cobalt alloy (Stellite 6) were studied. The experimental design method was a tool used for conducting their research. Predictive models concerning the evolutions of the arithmetic mean roughness, tangential force, the rate of stock removal and cutting power were established. Based on ANOVA—analysis of variance, and PARETO principle optimal cutting parameters for Stellite 6 were proposed. Sabri Ozturk [24] is another researcher who examined the machinability of Stellite 6 coating using a ceramic cutting tool. He successfully implemented the Taguchi experimental design to determine the optimal cutting parameters with which to analyse the effects of the cutting speed and feed rate on the surface quality. Sabri Ozturk [25] also estimated the turning conditions for Stellite 6 coatings in his other work. He tried to find the optimal cutting speed and feed rate in turning operations in terms of surface roughness using two different types of cutting tool: whisker-reinforced ceramic and tungsten carbide. It was found that a better surface finish could be achieved by low feed rates, and that feed rate had a higher impact on surface roughness than the cutting speed. Yingfei et al. [26] focused on the effect of cutting parameters on the surface and residual stress during all conditions in turning operations. They found that as tool wear increased, the surface roughness increased, and the deformation layer was found to be influenced more by the cutting parameters than by the tool wear. 

### 1.3. Machining of Coatings

According to the authors [27,28,29], the machining of coatings, especially after thermal spraying, has to address two main problems: the adhesion of the coating to the substrate and the wear of the tool. Moreover, the authors [30] state that the efficiency of the machining process depends on the structure and properties of the materials in contact. Achievement of the expected and desirable results, therefore, depends to a large extent on selected tool materials and selected machining parameters [31]. Adequate selection of machining parameters can help to achieve acceptable surface quality and tool wear. Typically, cutting rates of approximately 30–40 m·min^−1^ and relatively low values of cutting depth and feed rate are used [27]. Although machining is a complex process that is influenced by many factors, current studies have focused on selecting the machining parameters for turning operations. An empirical and mechanical approach to selecting machining parameters has been identified by the authors [32] for surface roughness analysis, although both approaches could be used for assessment of other outputs. The considerable complexity of the mechanical approach is simplified by the empirical approach. Therefore, several experimental tests with different machining parameters are usually performed to predict the final effect on the results. The empirical methodology is particularly suitable for machining processes, representing a strong background for stabilising the initial values of the machining parameters. However, this background is not large enough for machining of coatings, which are difficult to cut. Although the choice of machining parameters plays an important role in the process results, it should be emphasised that other factors can also have a significant impact. The influence of machining parameters is more accepted in the scientific literature than it is monitored by technical practice; in this sense, more results of the machining process are affected. In the case of hard turning, the authors [33] confirmed the influence of cutting rate, cutting depth, feed rate and machining times, surface roughness and wear of the tool. The effects of machining parameters on surface quality have been given by the authors [34], who evaluated the turning of sintered WC–25Co with the use of cutting tools by tests, which identified an unequivocal relationship between cutting rate and surface roughness. At cutting rates of 15 and 40 m·min^−1^, the surface roughness was limited to less than 0.2 μm for all machining times tested. However, at a cutting rate of 100 m·min^−1^, the surface roughness achieved significantly higher values and reduced the machining time.

On the other hand, the authors [35] found that the choice of feed rate (from 0.03 to 0.3 mm·rev^−1^) played an essential role in the development of surface roughness. In analysing the effect of cutting depth, it was found that the use of sharp-edged tools caused an increase in cutting depth and lower values of surface roughness. Based on the research conducted on Stellite 6 plasma transferred arc (PTA) coatings on a steel substrate and knowledge about the machinability of this hard coating, the authors [36] optimised the machining parameters through the surface roughness. Surface roughness values were examined at different cutting speeds, feed rates and depths of cut.

### 1.4. Modelling and Optimisation Techniques

There are many modelling and optimisation techniques that can be used in turning processes. Kumar et al. [37] published a review on modelling and optimisation methods in the turning process. This work discussed modelling and optimisation techniques, multi optimisation techniques, their applications and the advantages and limitations of them. With an appropriate model, showing the relationship between the input cutting conditions and the response of a system, an improved understanding of the process can be reached. Some researchers have focused on using regression-based modelling, artificial neural networks or fuzzy modelling. Aykut et al. [38] used artificial neural networks (ANNs) to model the effects of machinability on chip-removal cutting parameters for face milling of Stellite 6 in asymmetric milling processes. ANN has been proven to be an excellent tool for prediction of the asymmetric milling process. None of the previously published studies have focused on prediction of both material and technological parameters of the longitudinal turning of cobalt alloys using the mathematical–physical model. No authors have, so far, theoretically dealt with combining the coating machinability parameters (in particular, Stellite 6 applied to the base material) and the roughness parameters of the coating, i.e., by developing a prediction model for binding the machining technology parameters with the surface topography parameters.

For this reason, the present paper dealt particularly with a new method of predicting the material and technological parameters of longitudinal turning, so that the exceptional Stellite alloy might become more easily machinable and, therefore, available for other, more demanding applications. This model can be used in practice and research for the determination of optimum machining parameters (feed, cutting speed, depth of cut and others) through surface roughness, according to the pre-determined machinability of a specific material. 

## 2. Materials and Experimental Procedures

### 2.1. Material

**Figure 1:**
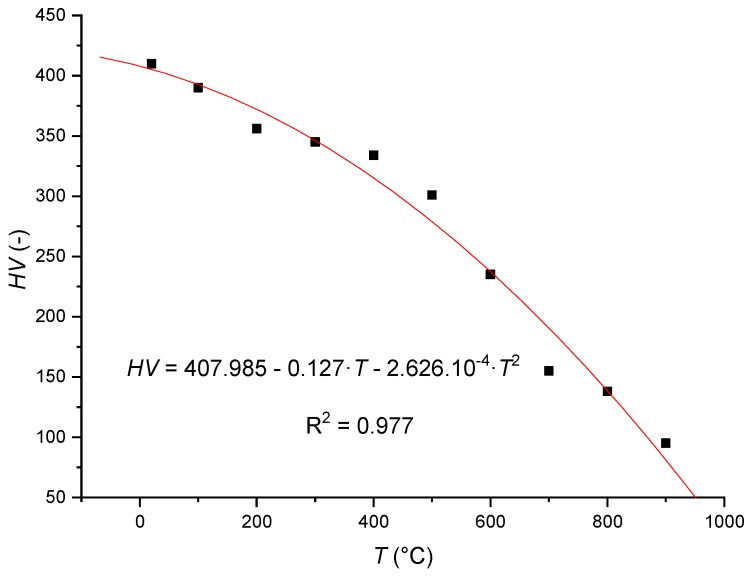
The nominal value of hardness of Stellite 6 spray in hot conditions.

Stellite 6 is the Co–Cr–W alloy is most widely used in industrial applications. When applying a layer of the Stellite 6 coating to a surface, different technologies can be used for its application, such as plasma transfer arc (PTA), inert tungsten gas (TIG) welding, thermal spraying or laser coating. The chemical composition of the Stellite 6 spray, its basic mechanical properties, the nominal values of the stress and the nominal values of hardness in hot conditions are given in Table 1 and Table 2 and Figure 1 [39]. Table 1 presents the specified mean composition values of Stellite 6 obtained from the scanning electron microscopy/energy dispersive X-ray spectroscopy (SEM/EDX, Carl Zeiss s.r.o., Prague, Czech Republic) measurements [40], with the density of the test material being 8.44 g cm^−3^.

Temperature changes of stress nominal values *E_mat_*, *Rp*_0.2_ and *Rm* of the core of the examined material have not been tabulated as dominant in the context of adequate changes of its surface, although, generally speaking, for the core material, the effect of heating on deformation under certain conditions (especially statically indeterminate structures) may exceed the effect of mechanical (tensile and compressive) forces. For the surface of the examined material, the change in surface hardness was observed as dominant in the context of temperature changes. Figure 1 describes a nonlinear downward trend in the nominal value of Stellite 6 spray in hot conditions [39]. 

### 2.2. HVOF Spraying Technology

The presented experiments used the spraying technology HP/HVOF JP500, implemented at the Research and Testing Institute in Plzeň, Ltd (Pilsen, Czech Republic). The parameters of spraying are summarised in Table 3.

**Figure 2:**
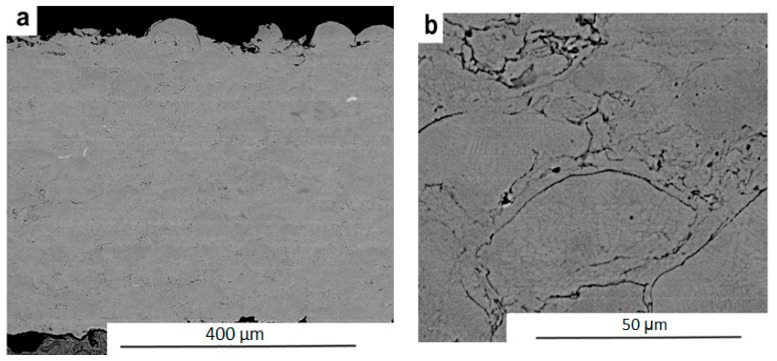
The scanning electron microscope (SEM) microstructure of Stellite 6 (Co-Cr-W alloy)) High pressure (HP)/High velocity oxy-fuel sprayed coating (HVOF) sprayed coating: (**a**) overview; (**b**) detail.

Before spraying, the surface of the component was prepared by aluminium oxide blasting with a grain size of 0.8–1 mm. Particles of the size 20–53 μm atomised by gas were used for spraying. The final surface roughness after blasting (before spraying with Stellite 6) was 8 μm. The resulting microstructure of the coatings is shown in Figure 2. 

Figure 2b exhibits a specific porosity in the coated area of less than 1%. If the porosity exceeded 1%, it would have been necessary to evaluate not only this parameter, but also the association with adhesion and possible oxidation in the formation of the surface layer. Higher porosity could significantly affect the mechanical behaviour, and hence the desired machinability of the material surface.

Within the frame of experimental verification, cylindrical samples with a diameter of 54.7 mm, made of the base material C45 were used, onto which Stellite 6 was applied by high-velocity oxygen fuel spraying with an average layer thickness of 0.55 mm, according to the conditions presented in Table 3. The length of the machined part of the sample was determined to be 88 mm. 

The machining was performed using the tool TUNGALOY RNGN 120400 LX11 43 (TUNGALOY CZECH s.r.o, Brno, Czech Republic), which was clamped in the tool holder MRGNR2525M12 (Kennametal Österreich GmbH, Perchtoldsdorf, Austria). 

**Figure 3:**
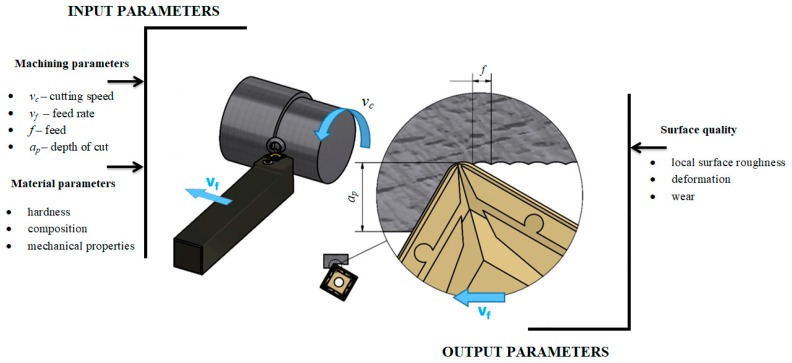
Diagram of Stellite 6 machining.

The actual experimental verification was performed on the general purpose centre lathe MASTURN 50/C80 (KOVOSVIT MAS Machine Tools, a.s., Sezimovo Ústí, Czech Republic, Figure 3) according to the conditions specified in Table 4 (where *f* is the feed, *v_c_* is the cutting speed and *a_p_* is the depth of cut). 

### 2.3. Surface Roughness Measurement

The morphology of the machined area was monitored by the contact profile meter HOMMEL-ETAMIC TURBO WAVE V7.45 (HOMMEL CS s.r.o., Teplice, Czech Republic) with the following settings:

- Sensor = TKU300;
- Measuring range = 800 μm;
- Measuring track *l_t_* = 4.80 mm;
- Feed rate *v_t_* = 0.50 mm·s^−1^.

The following parameters of surface roughness were selected as the basic indicators of the morphology of the machined surfaces as part of the experimental verification:

- Arithmetical centre of absolute deviations of the filtered roughness profile from the centre line within the basic measuring length *l_r_*—*Ra*;
- The measuring length corresponded to DIN EN ISO 4287 standard, i.e., five sections (*l_n_* = 5 × *l_r_*; *l_r_* = *λ_c_*), where half of the first section is for the touch sensor start-up, and half of the last section is for the touch sensor stop.

**Figure 4:**
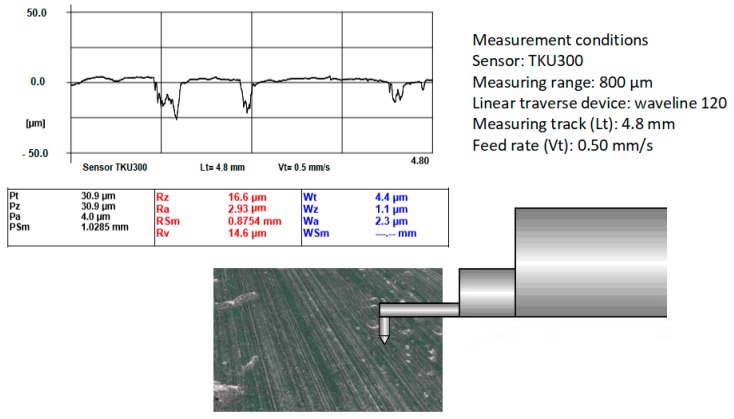
Measurement of surface roughness (real surface).

Figure 4 schematically shows how surface parameters were measured. The measured surface roughness data were compared and verified in the text below, see Equations (2) and (21). 

## 3. Results and Discussion

From the viewpoint of chip machining of Stellite 6 spray, several specific issues exist related to the properties of the spray that impair its machinability.

### 3.1. Attributes of More Difficult Machinability of Stellite 6

Machinability generally depends on the following [41,42,43,44,45]:

- Method of manufacturing and heat treatment of machined material,
- Microstructure of machined material,
- Chemical composition of machined material,
- Physical and mechanical properties of machined material,
- Methods of machining,
- Working environment,
- Tool geometry,
- Type and characteristics of the tool material.

Several methods are used for the objective assessment of machinability. The available cutting speed for the given conditions, the cutting resistance generated during machining, the input power required for machining, the cutting edge temperature, the quality of the machined surface and other parameters are monitored.

In the machine-building industry, the machinability of a given material is expressed by the ratio of the possible cutting rate for this material to the cutting rate possible for the standard material, under otherwise identical conditions.

Stainless steels listed in European standards are classified according to their metallographic structure and chemical composition into quality groups, and the reference material for the B group of steel grade is material 12 050.1 (or EN 10083-2-91). According to CNN (National Standards and Normatives), technical and construction materials are divided into nine groups. In each group, one particular material is labelled the standard (for steel it is 14 B, as a noble carbon steel 12 050.1), against which the relative machinability of all other materials in a given group is subsequently determined. The materials of each group are then further divided into individual classes of machinability based on the so-called index of kinetic machinability, which is commonly used in mechanical engineering (Equation (1)):

(1) $$i_{o}\;=\;\frac{v_{c15}}{v_{c15et}},$$

where *v_c_*_15_ is the cutting speed of the tool at its durability *T* = 15 min for the analysed material, and *v_c_*_15*et*_ is the cutting speed of the tool at its durability *T* = 15 min for the standard material. The standard material of a class of has a specific value of *i_o_* = 1. 

Individual classes of machinability are designated by the digits placed in front of the letter of the given group. Materials in classes with a lower number have worse machinability than those with a higher number. Individual classes are graded according to the average value of the machinability index, which for steel is *q* = 10(1/10) = 1.26. The cutting speed value *v_cT_* in the adjacent class is 1.26 times higher or lower [44,45]. 

Stellite 6 is a highly demanding alloy regarding chip machining technology. Thanks to its unique properties, it is a highly requested material [39].

Stellite 6 is a tough alloy, which leads to higher wear of tools and worse machinability. When comparing the modulus of elasticity in the tension of Stellite 6 and steel 11,373, we obtained the value *IND_e_* = 1.15 (ratio of the values 237,000/206,000). Tabular values of Young’s modulus of elasticity *E_mat_* for the coating and substrate were relatively close. The contents of the elements Co, Cr and W in the structure of Stellite 6 significantly increase its strength, hardness and toughness. This fact is demonstrated more distinctly by comparing the parameters of the yield strength *R_e_* or *R_m_*. The relative value is *IND_re_* = *Re/Re*(et) = 3.333 (ratio of the values 750/225), or *IND_rm_* = 3.549 (ratio of the values 1260/355). These values represent machinability of Stellite 6 up to 3.5 times that of the the base steel 11,373.

### 3.2. Analysis of the Investigated Parameter Ra—Statistical Model

Analysis of the investigated parameter *Ra* (Table 5) at a constant setting and action of the tool RNGN43-LX11-TUNGALOY indicated that variability of the investigated parameter *Ra* was 99.86%, with an average value of 1.98 μm. The selected dependence was, therefore, functional from the viewpoint of statistics. In the regression analysis, the coefficient of multivariate correlation R^2^ was chosen instead of the coefficient of determination R (using the Linregression function).

The adequacy of the chosen model was tested by a scattering analysis (Table 6) when testing a zero (*H*_0_) statistical hypothesis based on the nature of the test, which indicated that none of the effects used in the model affected the significant change of the investigated variable. It followed from the test that the obtained level of significance (Prob > F) was smaller than the chosen level of significance *α* = 0.05 and that it was, therefore, possible to accept the conclusion that we did not have enough evidence for accepting *H*_0_, and we were able to claim that the model was significant. Therefore, it can be assumed that the variability caused by random errors was significantly smaller than the variability of the measured values explained by the model.

On the basis of testing the adequacy of the statistical model used, it is possible to determine the regression coefficients of statistical dependence (Table 7).

The sought mathematical statistical dependence of the roughness parameter *Ra* on the cutting conditions can be expressed according to Equation (2):

(2) $$Ra=-1.116+7.700\cdot f+3.157\cdot 10^{-3}\cdot v_{c}-15.450\cdot a_{p}.$$

**Figure 5:**
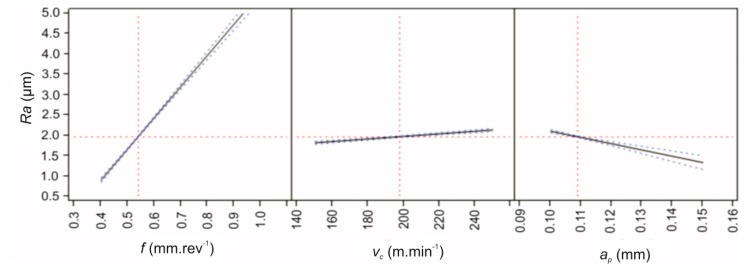
Prediction of the dependence of the parameter *Ra* on feed rate *f*, cutting speed *v_c_* and depth of the cut *a_p_*.

It follows from the statistical analysis and from Equation (2) that increasing the feed value also increased the conditional value of the investigated parameter *Ra*. The feed rate had a 69.55% effect on the change of the value of the *Ra* parameter. Similarly, increasing the cutting speed value resulted in an increase in the value of the *Ra* parameter. The cutting speed affected the change in the value of the *Ra* parameter from 17.25%. Increasing the depth of cut decreased the conditional value of the surface roughness *Ra*. The depth of cut had a 13.19% effect on the change in value of the *Ra* parameter. The influences of the individual cutting conditions on the change of the value of the *Ra* parameter are graphically illustrated in Figure 5.

**Figure 6:**
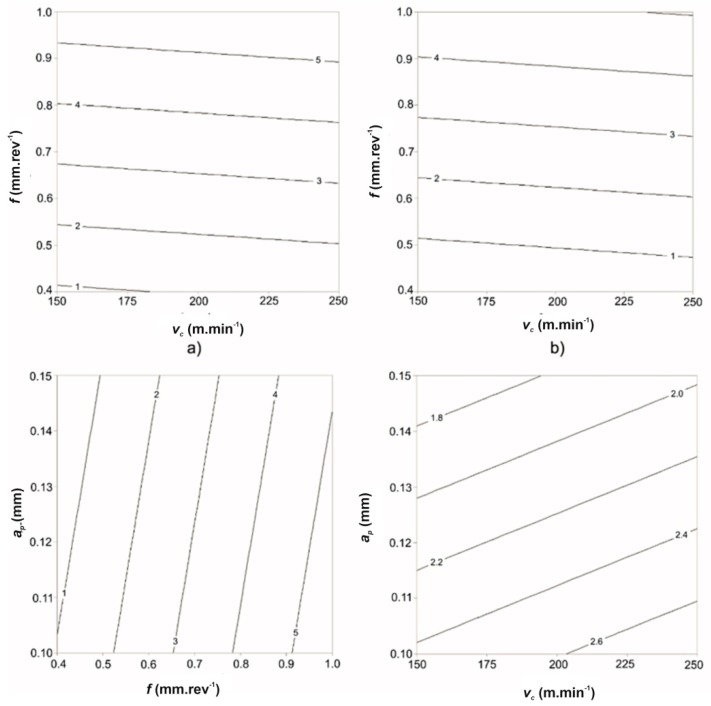
Dependence of change of the value *Ra* on the change of cutting conditions: (**a**) *a_p_* = 0.1 mm, (**b**) *a_p_* = 0.15 mm, (**c**) *v_c_* = 200 m·min^−1^, (**d**) *f* = 0.6 mm·rev^−1^.

From the developmental trends (Figure 6) and the previous analysis, it is clear that the feed rate, cutting speed and cutting depth influenced the value of the parameter *Ra*. In the range of the experimentally used cutting conditions (Table 4), it was possible to reach the minimum value of the investigated parameter *Ra* when using the lower limit of the cutting speed and the lower limit of the feed rate. In this area, the value of the parameter *Ra* was within 2 μm. This range of minimum values of *Ra* was within the interval of feed rates from 0.4 to 0.5 mm·rev^−1^. By increasing the depth of cut to *a_p_* = 0.15 mm, the area of minimal value of surface roughness *Ra* was moved to the interval of feed rates from 0.4 to 0.6 mm·rev^−1^ across the entire experimentally selected interval of cutting speeds. On the other hand, a small effect of the cutting speed was observed on the change of value of the parameter *Ra*.

### 3.3. Prediction of Parameters of the Longitudinal Turning Technology—Analytical Model

Based on the previously derived relationships [20,21], a comparison of the obtained data was made. Equation (3) is derived from the topography of split surfaces.

The presented Equation (3) declares a deformation constant *K_plmat_*:

(3) $$K_{plmat}\;=\;\frac{Ra\cdot a_{p}}{Y_{ret}},$$

where *a_p_* is the depth of cut and *Y_ret_* is cut trace retardation.

*K_plmat_* (μm) was determined in advance by measuring three deformation parameters of the sample of the material: selected cut depth *a_p_* (mm), local roughness *Ra_x_* (μm) at the depth *h_x_* and local deviation of the cut trace from the normal plane *Y_retx_* at the depth *h_x_*.

The empirical relationship of the deformation constant in direct dependence to *E_mat_* is shown by Equation (4):

(4) $$K_{plmat}\;=\;\frac{10^{12}}{E_{mat}^{2}}\;.$$

This is a widely applicable relationship in applications [46,47]. It is possible to define the following: “The product of roughness and depth divided by the deflection of the trace is the constant functionally bound to the *E_mat_* of the divided material” (Figure 5). It characterises the deformation capacity of the material. It also applies in an empirical relationship with the yield strength (Equation (5)):

(5) Re= Emat30.05·1012

For chip machining (longitudinal turning), it is possible to use new forms related to the depth of cut *a_p_*. Thus, we obtained Equations (6) and (7). The feed rate *f* = *f* (*Ra*) is given by Equation (7). Additionally, the feed rate of optimal *v_copt_* can be broken down into Equation (8), defining the relationships between the parameters of the cut and the parameters of the topography of the final surface:

(6) $$Ra\;=\;K_{plmat}\cdot \frac{Y_{ret}}{a_{p}},$$

(7) $$f=8.51.10^{3}.\frac{Ra}{\sqrt{E_{mat}}}$$

Equation (9) applies to the tool speed, where *Ra_o_* is surface roughness in the neutral plane of the cut:

(8) $$v_{copt}=\sqrt{10^{-3}\cdot Ra_{o}}\cdot \frac{10^{6}}{\sqrt{E_{mat}}}$$

as well as Equation (10), where, after substituting in Equation (5), we get the direct dependence *v_copt_* = *f* (*Re*):

(9) $$v_{copt}=\sqrt{10^{-3}\cdot Ra_{o}}\cdot \frac{10^{6}}{\sqrt{{\left(R_{e}^{2}\cdot \left(0.05\cdot 10^{12}\right)\right)}^{0.333}}}$$

The feed rate of the cutting tool can also be selected as a controlled speed to achieve the required roughness and a straight track cut without curvature. The control rate *v_cREG_* maintains a constant strain in the cutting walls along the entire depth, and, depending on the type of regulated roughness, it can be expressed by Equations (10)–(12):

(10) $$v_{cREG}=\sqrt{10^{-3}\cdot Ra_{o}}\cdot \frac{10^{6}}{\sigma_{rz}},$$

(11) $$v_{cREG}=\sqrt{10^{-3}\cdot Ra_{o}}\cdot \frac{10^{6}}{\sigma_{rad}},$$

(12) $$v_{cREG}=\sqrt{10^{-3}\cdot Ra_{o}}\cdot \frac{10^{6}}{\sigma_{sk}}$$

where *σ_rz_* is the pressure component according to Equation (13), *σ_rad_* is the tensile component according to Equation (14) and *σ_sk_* is the total stress according to Equation (15):

(13) $$\sigma_{rz}=10^{-3}\cdot Ra\cdot \frac{E_{mat}}{Ra_{o}},$$

(14) $$\sigma_{rad}=10^{-3}\cdot Ra_{d}\cdot \frac{E_{mat}}{Ra_{do}},$$

(15) $$\sigma_{sk}=10^{-3}\cdot Ra_{sk}\cdot \frac{E_{mat}}{Ra_{do}}$$

*Ra_sk_* is the roughness of cut trace at the depth according to Equation (16), radial roughness *Ra_d_* as given by Equation (17) is modified from Equation (16), radial roughness *Ra_do_* at the neutral plane is given by Equation (18) and *σ_pr_* is the tensile modular ratio between the tensile modular component *E_ret_* and the Young’s modulus *E_mat_*, according to Equation (19):

(16) $$Ra_{sk}=10^{{\left(\log a_{p}\right)}^{2}+\sqrt{ĺog{\left(\sigma_{pr}\right)}^{2}+Ra_{rad}^{2}}},$$

(17) $$Ra_{d}=Ra_{o}\cdot 10^{3}\cdot \sqrt{\frac{E_{retz}}{E_{mat}}},$$

(18) $$Ra_{do}=Ra_{o}\cdot \frac{10^{3}}{E_{mat}},$$

(19) $$\sigma_{pr}=\frac{E_{ret}}{E_{mat}}.$$

The pressure modular component is then given by the evolution of the *E_retz_* curve according to Equation (20). The tensile modular component is given by the evolution of the *E_ret_* curve according to Equation (20), below:

(20) $$E_{retz}=E_{mat}\cdot \sqrt{\frac{Ra\cdot a_{p}}{K_{plmat}}}.$$

The regulation speed (Equation (12)) prevents the local increase of the analytical roughness *Ra_A_* at the penetration of the tool into the material. For a generally chosen feed rate *v_c_*, a simple proportional relationship between the chosen speed *v_c_* and the optimal speed *v_c_*/*v_copt_* can be used, as shown by Equation (21):

(21) $$Ra_{A}=Ra_{o}\cdot \sqrt[{\frac{3}{2}}]{\frac{v_{c}}{v_{copt}}}.$$

For *E_ret_* (Equation (22)) and *a_pc_* (Equation (23)), the following applies:

(22) $$E_{ret}=E_{mat}\cdot \sqrt{\frac{K_{plmat}}{Ra\cdot a_{p}}},$$

(23) $$a_{pc}=Ra\cdot \frac{a_{p}}{K_{plmat}}.$$

### 3.4. Comparison of the Mathematical Statistical and Analytical Approaches

**Figure 7:**
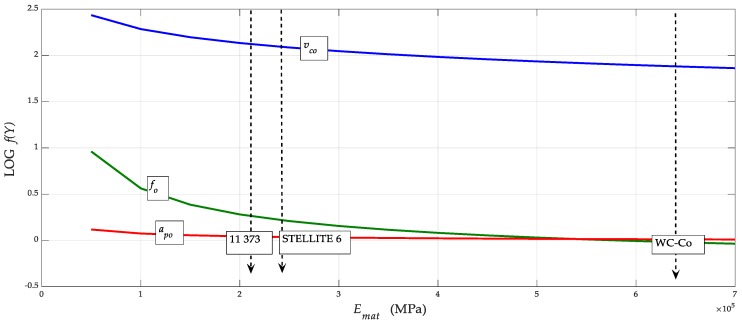
Dependences (*v_co_*, *a_po_*, *f_o_*) = *f* (*E_mat_*) for Stellite.

**Figure 8:**
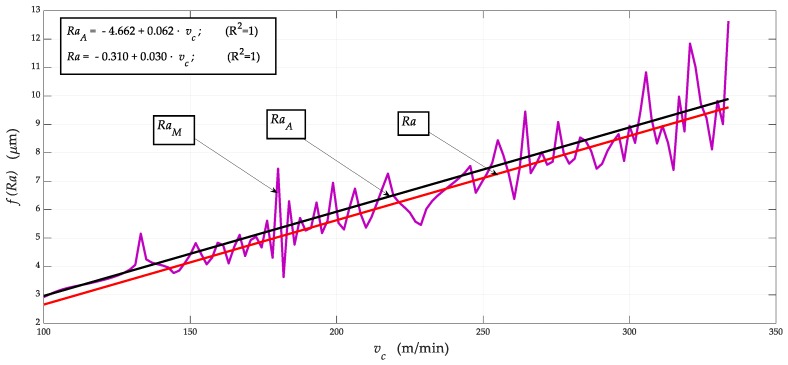
Comparison of (*Ra_M_*, *Ra_A_*, *Ra*) = *f*(*v_c_*) according to values in the columns 7, 8 and 9 in Table 8; *Ra_M_* measurement data, *Ra_A_* according to Equation (21), above, and *Ra* according to Equation (2).

**Figure 9:**
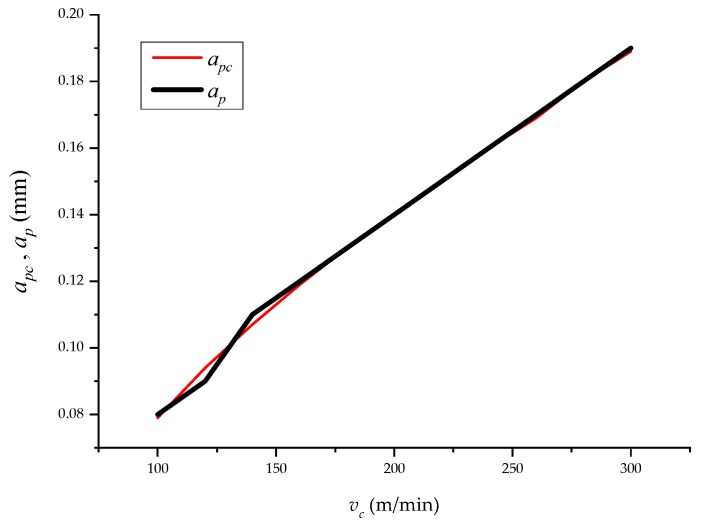
Comparison of (*a_p_*, *a_pc_*) = *f* (*v_c_*) according to values in columns 3 and 12 in Table 1; *a_p_* chosen, *a_pc_* calculated for control according to Equation (23), below.

Based on analysis of experimentally obtained data, a comparison of the mathematical statistical and analytical approaches is presented. Table 8 and Figure 7, Figure 8 and Figure 9 present the selected technological parameters (longitudinal turning parameters) *v_c_*, *f* and *a_p_* for the experimental combinations. Table 8 follows and presents the pre-selected values of parameters, which were determined analytically. 

For analytical and, of course, practical interpretation of the values in the tables, the authors derived the basic equations of equilibrium for conventional machining in Equations (3) to (31). In deriving these new equations, the main emphasis was put on the basic mechanical parameters of the material being machined. These equations are based on the considerations and relations given above in Section 3.3 and from patented solutions [17,18]. As can be seen from the composition of the equations, the basic equation of equilibrium for chip machining is Equation (3). It is possible to continue and to supplement these equations with some other derived equations for other desired applications.

### 3.5. Equation for Machining Parameters R_mp_

The new parameter *R_mp_* (no unit) was derived by the authors in order to provide a comprehensive and simple quantification. This physically discrete equation of machining parameter *R_mp_* has been generally derived for materials in Equations (24)–(28):

(24) $$R_{mp}=\left(\frac{f}{f_{o}}\cdot \frac{v_{c}}{v_{co}}\cdot \frac{a_{p}}{a_{po}}\right).$$

Equation (25) applies to the neutral plane in machining:

(25) $$R_{mpo}=\left(\frac{f_{o}}{f_{o}}\cdot \frac{v_{co}}{v_{co}}\cdot \frac{a_{po}}{a_{po}}\right)=1.$$

For analytical calculation of the roughness generated in machining, Equations (26) and (27) apply:

(26) $$Ra_{mp}=k_{mes}\cdot Ra_{o}\cdot \left(\frac{f}{f_{o}}\cdot \frac{v_{c}}{v_{co}}\cdot \frac{a_{p}}{a_{po}}\right),$$

(27) $$Ra_{mp}=k_{mes}\cdot Ra_{o}\cdot R_{mp}.$$

For analytical calculation of the roughness at the neutral plane generated in machining, Equation (28) applies:

(28) $$Ra_{mpo}=k_{mes}\cdot Ra_{o}\cdot \left(\frac{f_{o}}{f_{o}}\cdot \frac{v_{co}}{v_{co}}\cdot \frac{a_{po}}{a_{po}}\right)=3.7.$$

In the physically discrete equations, and in addition to the above, the following parameters are defined:

*f_o_*—feed rate at the neutral plane *h_o_* (mm∙rev^−1^);
*v_co_*—speed at the neutral plane *h_o_* (m/min);
*a_po_*—depth of cut at the neutral plane *h_o_* (mm);
*Ra_o_*—surface roughness at the neutral plane *h_o_* (μm);
*k_mes_*—constant according to laboratory conditions for measurement of roughness *Ra*, vibration (no unit).

The parameters for the neutral plane can be determined by the following Equations (29) to (31):

(29) $$v_{co}\;=\;\sqrt{10^{-3}\cdot Ra_{o}}\cdot \frac{10^{6}}{\sqrt{E_{mat}}},$$

(30) $$a_{po}=2\cdot \left(-\;0.405\;+\;0.005\cdot v_{popt}\right),$$

(31) $$f_{o}=\frac{8.51\cdot 10^{3}\cdot Ra_{o}}{\sqrt{E_{mat}}}.$$

For better clarity, and especially for the possibility of directly reading the parameters at the neutral level, a graphical representation can be constructed, using the generalised dependence (*v_co_*, *a_po_*, *f_o_*) = *f* (*E_mat_*, *Re*) according to the equations derived by the authors. Equation (30) can be adapted to the form *a_po_* = *f* (*E_mat_*) by, for example, using Equation (31). A constructed graph for fast reading of neutral parameters *v_co_*, *a_po_* and *f_o_* is shown in Figure 7. The data for *v_co_* in the graph are given in decimal logarithms due to the variance of the values, and must be converted to a natural number after deduction from the graph. If the accuracy requirement is stricter, the calculation can be made according to Equations (29)–(31).

If we have determined the *R_mp_* value according to Equation (24) for the given combination of main machining parameters, *v_c_*, *f* and *a_p_*, the *Ra* roughness values can be calculated discretely using *R_mp_*, including determination of the influence/proportion of each parameter on the surface roughness. For machined materials in general, Equations (32)–(34) apply:

(32) $$v_{c}=v_{co}\cdot \frac{R_{mpo}}{\left(\frac{f}{f_{o}}\cdot \frac{a_{p}}{a_{po}}\right)},$$

(33) $$f=f_{o}\cdot \frac{R_{mpo}}{\left(\frac{v_{c}}{v_{c}{}_{o}}\cdot \frac{a_{p}}{a_{po}}\right)},$$

(34) $$a_{p}=a_{po}\cdot \frac{R_{mpo}}{\left(\frac{f}{f_{o}}\cdot \frac{v_{c}}{v_{co}}\right)}.$$

Based on the ratio (Figure 8) between the selected velocity *v_c_* and the velocity at the neutral plane *v_o_* = *f*(*h_o_*), *Ra* can be generally obtained with certainty and in a very simple way at any *h*. 

Comparing our two models, and based on Equations (2) and (21), it was possible to determine the tightness of conformity R^2^ = 1 with the measured data in both models.

This confirmed that the speed *v_c_* had a significant influence on the surface roughness *Ra*. Similarly, the *a_p_* ratio can be calculated from the rate ratio (Figure 9).

**Figure 10:**
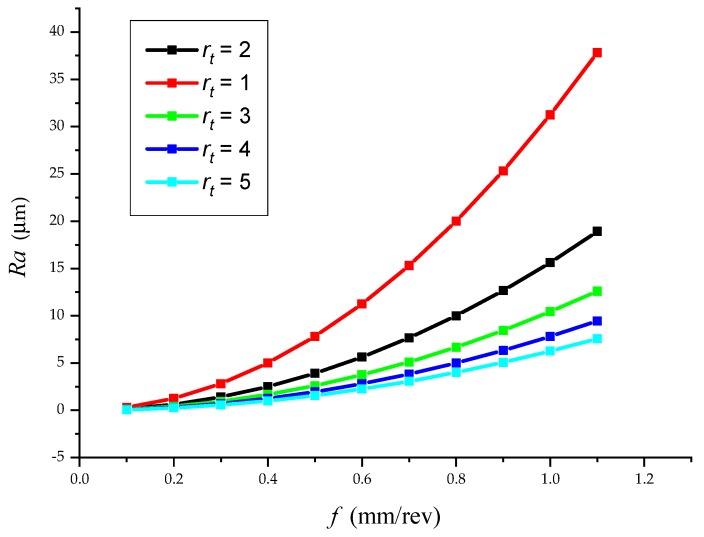
The effect of blunting the tool edge *r_t_* on the surface roughness *Ra* = *f* (*f*).

**Figure 11:**
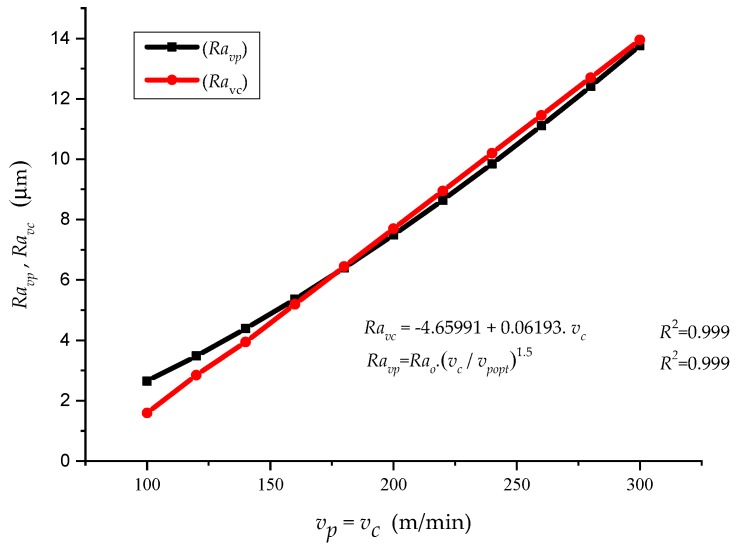
Comparison of (*Ra_vp_*, *Ra_vc_*) = *f* (*v_c_*).

Figure 10 and Figure 11 present comparisons between individual mathematical statistical and analytical approaches, as well as the comparisons from the measurements and the new prediction equations.

Roughness *Ra* (Figure 9) was calculated according to the modified Equation (35):

Feed rate *f* was calculated for the control by modification from Equation (35) according to Equation (36):

(35) $$Ra=10^{3}\cdot 0.25\cdot \frac{f^{2}}{8\cdot r_{t}}.$$

Feed rate *f* was calculated for control by modification from Equation (35) according to Equation (36):

(36) $$f=Ra\cdot \frac{8\cdot r_{t}}{\sqrt{10^{3}\cdot 0.25}}.$$

**Figure 12:**
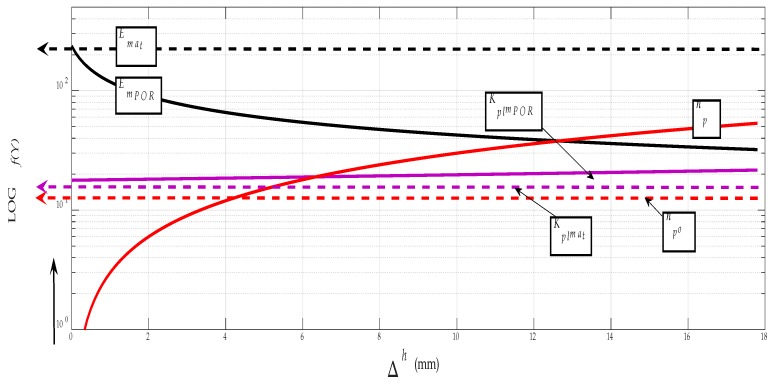
Porosity (*n_pX_*) = *f* (Δ*h*) for Stellite.

Figure 12 shows comparisons (*Ra_vp_*, *Ra_vc_*) = *f*(*v_p_*, *v_c_*). The equations describe individual dependences, with correlation leakage R = 0.999 that are represented by Equations (37) and (38):

(37) $$Ra_{vc}=-4.65991+0.06193\cdot v_{c}.$$

(38) $$Ra_{vp}=Ra_{o}\cdot \sqrt[{\frac{3}{2}}]{\frac{v_{c}}{v_{popt}}}.$$

### 3.6. The Influence of Porosity on Strength

**Figure 13:**
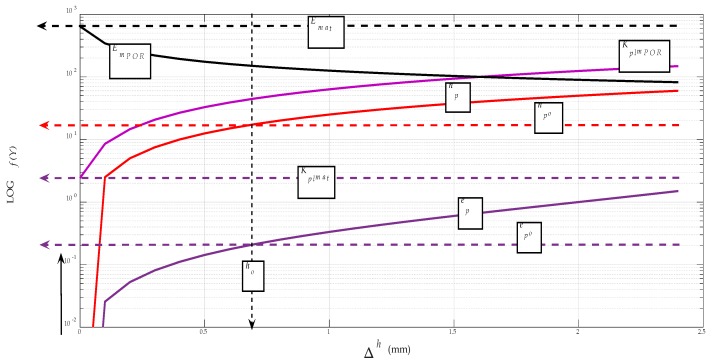
Porosity (*n_pX_*) = *f* (Δ*h*) for WC–Co.

The percentage of porosity in the baseline (coated) microstructure has a significant influence on the changes in the mechanical and adhesive parameters of the carbide coating cemented onto the substrate (Figure 12 and Figure 13). This applies to materials in general. To generalize this effect, we can fully use our parameter for material deformation length *K_plmat_* according to Equation (8) by adding to Equation (39), where *n_p_* = volume porosity according to:

(39) $$n_{p}=\;\frac{V_{por}}{V_{c}}\cdot 100.$$

where *V_por_* = pore volume and *V_c_* = total volume.

The deformation length extension constant according to the porosity *K_plmPOR_* can be determined according to Equation (40):

(40) $$K_{plmPOR}=\;\frac{K_{plmat}}{0.01\cdot n_{por}}.$$

The reduction of the Young’s modulus is also given by adjusting Equation (4) to Equation (41):

(41) $$E_{mPOR}=\frac{10^{12}}{\sqrt{K_{pl_{mPOR}}}}.$$

Porosity according to Equation (42) can also be used in the calculations:

(42) $$e_{p}=\frac{n}{\left(1-n\right)}.$$

where *e_p_* = space/volume porosity number and *n* = total porosity in %.

After such adjustments, the porosity volume ratio can generally be included in all material and application calculations, making full use of the authors’ patented algorithms [7]. According to these, the diagrams of interest parameters are shown below, both for Stellite spraying and for the possibility of comparing the very strong WC–Co carbide. The graphs are plotted according to the extension of the deformation length Δ*h*, analogously, they can also be plotted according to the relative elongation *ε*, or according to the deformation time *t_def_* (s). The graphs show the regularity when the deformation length of material *K_plmPOR_* is prolonged due to porosity, and, thus, the value of elasticity modulus *E_mPOR_* reduces, as well as the coating/spraying strength and tensile stress, together with yield strength *Re* or specific weight *ρ_o_*.

The paper presents mathematical statistical and analytical approaches and their comparison based on experimentally measured data, i.e., the mathematical statistical model (Equation (2)) was derived from the experimentally measured data. From analysis of the experimentally obtained data (Table 8), newly derived analytical equations were obtained, including the material parameters of the workpiece (Equations (5)–(8) and (23)–(42)). The significance of these relations lies in the fact that they include all three basic parameters of chip machining, *v_c_*, *f*, *a_p_*, and express the physical–mechanical parameters of the machined material.

## 4. Conclusions and Future Works

The presented paper aimed to propose a new, original method for prediction of material and technological parameters of longitudinal turning. Based on experimental data, it was possible to predict an exact determination of technological parameters of longitudinal turning using the newly proposed method. With increasing intensity of mechanical stress by longitudinal turning, the surface roughness of the material decreased, and vice versa. When a user assesses the required roughness of the workpiece surface, they are primarily concerned with maintaining the quality of the material and its functional service life.

The main assets of the paper can be summed up in the following points:

- A mathematical statistical model (Equation (2)) of the investigated *Ra* parameter was created from analysis of the experimentally obtained data;
- New analytical equations were created from analysis of experimentally obtained data, which serve to predict parameters of longitudinal turning technology (Equations (3)–(42));
- Newly derived analytical equations were obtained from the experimentally obtained data (Table 8), which include the material parameters of the workpiece;
- The comparison of the mathematical statistical and analytical approaches, and their verification with the experimentally measured data, confirmed the correspondence of both these approaches;
- The original relationships include in their calculations all three basic parameters of chip machining, *v_c_*, *f* and *a_p_*, and they also contain the physical–mechanical parameters of the machined material. This enables precise optimisation of the technological parameters of the turning according to the actual mechanical parameters of the material being turned;
- By deriving the new parameter *R_mp_* (Equation (25)) and others, as given in Section 3.5, the discrete influences of the main parameters, *v_c_*, *f* and *a_p_*, according to the above-presented Equations (35)–(37) can be calculated.

During spraying, undesirable structural defects such as porosity (40) to (42), oxidic phases or unstable amorphous phases can form in the coating structure. If spraying parameters are not found to minimize these undesirable defects, they should be taken into account when machining. Based on the presented method of prediction of parameters of longitudinal turning technology, the user’s primary interest is to determine the optimum values at a given quality and surface roughness to be machined, and especially the recommendation of suitable technological parameters such *v_c_* (m∙min^−1^), *f* (mm∙rev^−1^) and *a_p_* (mm) in order to avoid poor quality during machining, or even tearing of the Stellite 6.

Further experimental research will be focused on monitoring the dependence of hardness on temperature, using a microhardness tester LECO DM AMH 55 (Type B evaluation of measurement uncertainty) according to the Vickers method. The Vickers Hardness (HV) shall be determined at a load of 30 g, with a duration of 15 s, by ASTME384 (Type A evaluation of measurement uncertainty). Additionally, the influence of porosity by metallographic tests, adhesion and formation of oxide layers will be investigated, because information about the volume fraction, distribution and morphology of individual phases/microstructural components, as well as data about their chemical compositions, are crucial for a comprehensive description of the structural parameters of materials. These comprehensive data will allow us to incorporate new parameters into more accurate prediction models.

## Figures and Tables

**Table 1:** Chemical composition values determined by energy dispersive X-ray spectroscopy (EDX) measurement and verified as identical to nominal composition (mass %).

	Co	Cr	W	Mo	Si	Mn	Ni	Fe	C
Wt. %	59.68	28.34	4.1	1.69	1.23	0.27	1.66	0.79	2.24

**Table 2:** Nominal values of stress at room temperature of the Stellite 6 spray.

	*E_mat_* ^1^	*Rp*_0.2_^1^	*Rm* ^1^	*ε* ^1^
**Stellite 6**	237 GPa	750 MPa	1265 MPa	4%

^1^*E_mat_*—Young’s modulus of elasticity, *Rp*_0.2_—yield strength, *Rm*—material strength, *ε*—relative elongation.

**Table 3:** Spray parameters.

O_2_	Fuel	Barrel Length	Spray Distance	Traverse Speed	Feed Rate	Carrier Gas N_2_	Offset	Number of Passes
996 L·min^−1^	277 L·h^−1^	150 mm	360 mm	250 mm·s^−1^	46 g·min^−1^	6.5 L·min^−1^	6	7

**Table 4:** Conditions of experimental verification.

TEST	TOOL	*v_c_* (m·min^−1^)	*f* (mm·rev^−1^)	*a_p_* (mm)	Machining Length (mm)
1	RNGN43-LX11-TUNGALOY	150	1	0.15	20
2	RNGN43-LX11-TUNGALOY	150	1	0.15	20
3	RNGN43-LX11-TUNGALOY	150	1	0.15	20
4	RNGN43-LX11-TUNGALOY	150	1	0.15	20
5	RNGN43-LX11-TUNGALOY	150	0.6	0.1	15
6	RNGN43-LX11-TUNGALOY	150	0.6	0.1	15
7	RNGN43-LX11-TUNGALOY	150	0.6	0.1	15
8	RNGN43-LX11-TUNGALOY	150	0.6	0.1	15
9	RNGN43-LX11-TUNGALOY	150	0.4	0.1	15
10	RNGN43-LX11-TUNGALOY	150	0.4	0.1	15
11	RNGN43-LX11-TUNGALOY	150	0.4	0.1	15
12	RNGN43-LX11-TUNGALOY	150	0.4	0.1	15
13	RNGN43-LX11-TUNGALOY	250	0.4	0.1	15
14	RNGN43-LX11-TUNGALOY	250	0.4	0.1	15
15	RNGN43-LX11-TUNGALOY	250	0.4	0.1	15
16	RNGN43-LX11-TUNGALOY	250	0.4	0.1	15

**Table 5:** Summary of the fit parameter *Ra*.

Indicator	*Ra* μm
Rsquare (R^2^)	0.998796
RSquare Adj (R^2^Adj)	0.998606
Root Mean Square Error (RMSE)	0.051968
Mean of Response	1.980435 μm
Observations or Sum Wgts (analysis of variance)	23
AICc (information criterion)	−61.6212
BIC (information criterion)	−59.4731

**Table 6:** Analysis of variance parameter *Ra*.

Source	DF	Sum of Squares	Mean Square	F Ratio	Prob > F
Model	3	42.57278	14.1909	5254.502	<0.0001
Error	19	0.051314	0.0027		
C. Total	22	42.6241			

**Table 7:** Parameter estimates of the investigated dependence for the variable *Ra*.

Term	Estimate	Std Error	*t* Ratio	Prob > |*t*|	Lower 95%	Upper 95%
Intercept	−1.11602	0.119268	−9.36	<0.0001 *	−1.36565	−0.86639
*f* (mm·rev^−1^)	7.7	0.183736	41.91	<0.0001 *	7.315436	8.084564
*v_c_* (m·min^−1^)	0.003157	0.000303	10.4	<0.0001 *	0.002522	0.003792
*a_p_* (mm)	−15.45	1.944481	−7.95	<0.0001 *	−19.5198	−11.3802

* Significant at the level of significance *α* = 0.05.

**Table 8:** Parameter estimates of the investigated dependence for the variable *Ra*.

*v_c_*	*f*	*a_p_*	*E_mat_*	*K_plmat_*	*v_popt_*	*Ra_M_*	*Ra_A_*	*Ra*	*a_pc_*	*Y_ret_*	*R_mp_*
(m·min^−1^)	(mm·rev^−1^)	(mm)	(GPa)	(μm)	(m·min^−1^)	(μm)	(μm)	(μm)	(mm)	(mm)	-
100	0.1	0.08	237	17.8	125	2.93	2.96	2.66	0.08	0.01	0.03
120	0.2	0.09	237	17.8	125	3.54	3.552	3.25	0.09	0.02	0.07
140	0.3	0.11	237	17.8	125	3.99	4.144	3.85	0.11	0.03	0.16
160	0.4	0.12	237	17.8	125	4.18	4.736	4.44	0.12	0.04	0.26
180	0.5	0.13	237	17.8	125	6.27	5.328	5.04	0.13	0.05	0.40
200	0.6	0.14	237	17.8	125	6.14	5.92	5.63	0.14	0.06	0.57
220	0.7	0.15	237	17.8	125	5.82	6.512	6.22	0.15	0.07	0.78
240	0.8	0.16	237	17.8	125	7.21	7.104	6.82	0.16	0.09	1.04
260	0.9	0.17	237	17.8	125	7.43	7.696	7.41	0.17	0.11	1.34
280	1	0.18	237	17.8	125	7.55	8.288	8	0.18	0.13	1.70
300	1.1	0.19	237	17.8	125	8.36	8.88	8.6	0.19	0.15	2.12
200	0.6	0.14	237	17.8	125	2.93	2.96	2.66	0.14	0.07	0.77

Comments on Table 8: The selected technological parameters *v_c_*, *f*, *a_p_*; Young’s modulus of elasticity of Stellite *E_mat_* (GPa); *K_plmat_* material deformation constant of Stellite (μm) according to Equation (3); Optimal sliding cutting speed for Stellite *v_popt_* (m/min) according to Equation (9); Measured value of surface roughness *Ra_M_* = *f*(*v_c_*, *f*, *a_p_*) according to Equation (9) in the article; Analytical value of surface roughness *Ra_A_* = *f*(*_opt_*, *E_mat_*, *v_c_*) according to Equation (22); Surface roughness from the cutting speed *Ra_vc_* = *f*(*K_plmat_*, *Y_ret_*, *a_p_*) for control, according to Equation (22); Theoretical depth of cut *a_pc_* = *f* (*E_mat_*, *Y_ret_*, *Ra*_A_) for control, according to Equation (23); Deviation from the track *Y_ret_* = *f* (*E_mat_*, *a_p_*, *Ra_A_*) for control, according to Equation (23);Calculation of physically discrete parameter of *R_mp_* is based on Equation (24).
